# IX Year

**Published:** 1873-04

**Authors:** 


### IX YEAR.

The simple fact that the Bistoury begins with this number the ninth year of its existence, is sufficient evidence not only that it is well supported by the public, but also the further fact, that such a journal is needed and therefore appreciated. No physician of a progressive nature, can afford to be without the Bistoury. It is invaluable to him, keeping him thoroughly apprised of what is transpiring in the medical sciences, without subjecting him to long and wearisome dissertations which he has neither time nor inclination to read. The Bistoury comes to him with its Medical Items and News, the current medical literature of the day, so epitomized as to be at once comprehensive and instructive. These selections are carefully made, rejecting everything of a visionary or hypothetical nature, confining our items to plain facts, and our formulæ to those of undoubted merit, and to those only recommended by physicians of unquestioned ability. We bestow much care and labor upon this department, making it the principal feature of the journal. That this department in the Bistoury is appreciated by the medical profession, we are assured, for we are daily in receipt of orders for it from physicians from all parts of the United States.

Our non-professional readers are attended to and interested by our contributors, our Household Hints and Recipes, and, we hope, by the editorial matter which we prepare for them. We receive many complimentary notices from our brethren of the press, for our journal, which are given voluntarily, without solicitation from printed slips or other tricks, for inveigling newspaper men into recognizing our peculiar claims for an "editorial notice" in their columns. We publish the BISTOURY every quarter, on time,—take it or not—as you choose; ir you do not, you are the loser and not us.

